# Supplementary material for: Highly Sensitive and Specific Detection of Mobilized Colistin Resistance Gene mcr-1 by CRISPR-Based Platform
Source: Microbiol Spectr. 2022 Aug 31;10(5):e01884-22. doi: 10.1128/spectrum.01884-22 (PMC9602551; doi:10.1128/spectrum.01884-22)

TABLE S1. The consistency comparison of RPA-CRISPR/Cas12a technology with PCR method.

	mcr-1-PCR positive	*mcr-1*-PCR negative	
RPA-CRISPR/Cas12a positive	15	0	[a]PPV=100%
RPA-CRISPR/Cas12a negative	0	55	[b]NPV=100%
	sensitivity=100%	specificity=100%	

[a]PPV, positive predictive value; [b]NPV, negative predictive value.

Figure S1 Verification of the *mcr-1* RPA assay by using agarose gel electrophoresis. The samples were detected: (1) *mcr-1*-positive *Escherichia fergusonii* (WH-ZX154); (2) IMP-26-producing *Enterobacter hormaechei* (WHCDC-SP67); (3) qnrS1-producing *Enterobacter cloacae* (WHCDC-XW38); (4) distilled water.

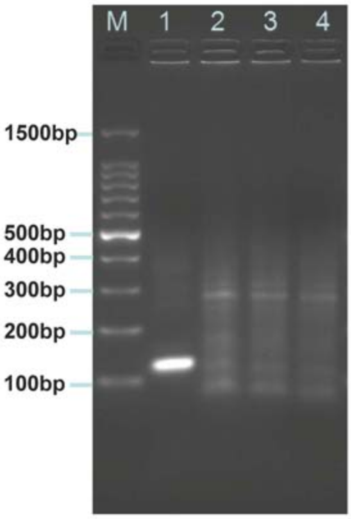

Figure S2 The LoD of the RPA-CRISPR/Cas12a system in *mcr-1*-spiked blood specimens. The results of three duplications were separately showed in Figures (A), (B) and (C). Table（D）showed the signal number matching each bacterial concentration and control. Signals 1-8 represented the reference strain DNA levels of 2.6×10^{3}, 2.6×10^{2}, 2.6×10^{1}, 2.6×10^{0}, 2.6×10^{-1}, and 2.6×10^{-2} CFU per reaction, non-spiked blood DNA, and distilled water, respectively.

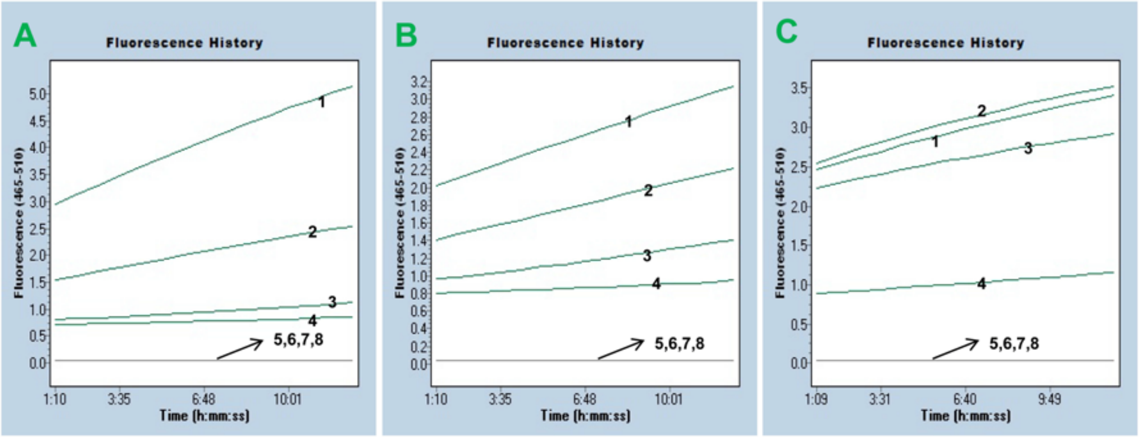

D

Signal numbers	1	2	3	4	5	6	7	8
Bacterial DNA levels (CFU/reaction)	2.6×10^{3}	2.6×10^{2}	2.6×10^{1}	2.6×10^{0}	2.6×10^{-1}	2.6×10^{-2}	non-spiked blood DNA	distilled water

Figure S3 The LoD of the RPA-CRISPR/Cas12a system in *mcr-1*-spiked urine specimens. The results of three duplications were separately showed in Figures (A), (B) and (C). Table（D）showed the signal number matching each bacterial concentration and control. Signals 1-8 represented the reference strain DNA levels of 1.6×10^{3}, 1.6×10^{2}, 1.6×10^{1}, 1.6×10^{0}, 1.6×10^{-1}, and 1.6×10^{-2} CFU per reaction, non-spiked urine DNA, and distilled water, respectively.

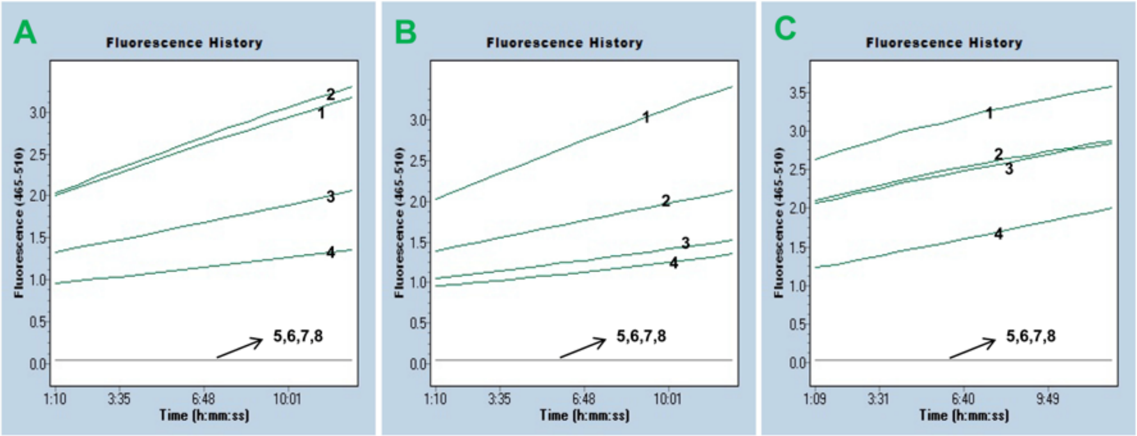

D

Signal numbers	1	2	3	4	5	6	7	8
Bacterial DNA levels (CFU/reaction)	1.6×10^{3}	1.6×10^{2}	1.6×10^{1}	1.6×10^{0}	1.6×10^{-1}	1.6×10^{-2}	non-spiked urine DNA	distilled water

Figure S4 Nucleotide sequence alignment of the *mcr-1* gene in various strains used to design the RPA primers and gRNA. The nucleotide sequence of the sense strand of *mcr-1* DNA is displayed. The sequence of the primer sites and gRNA sites are underlined, right and left arrows indicate sense and complementary sequences that are used.

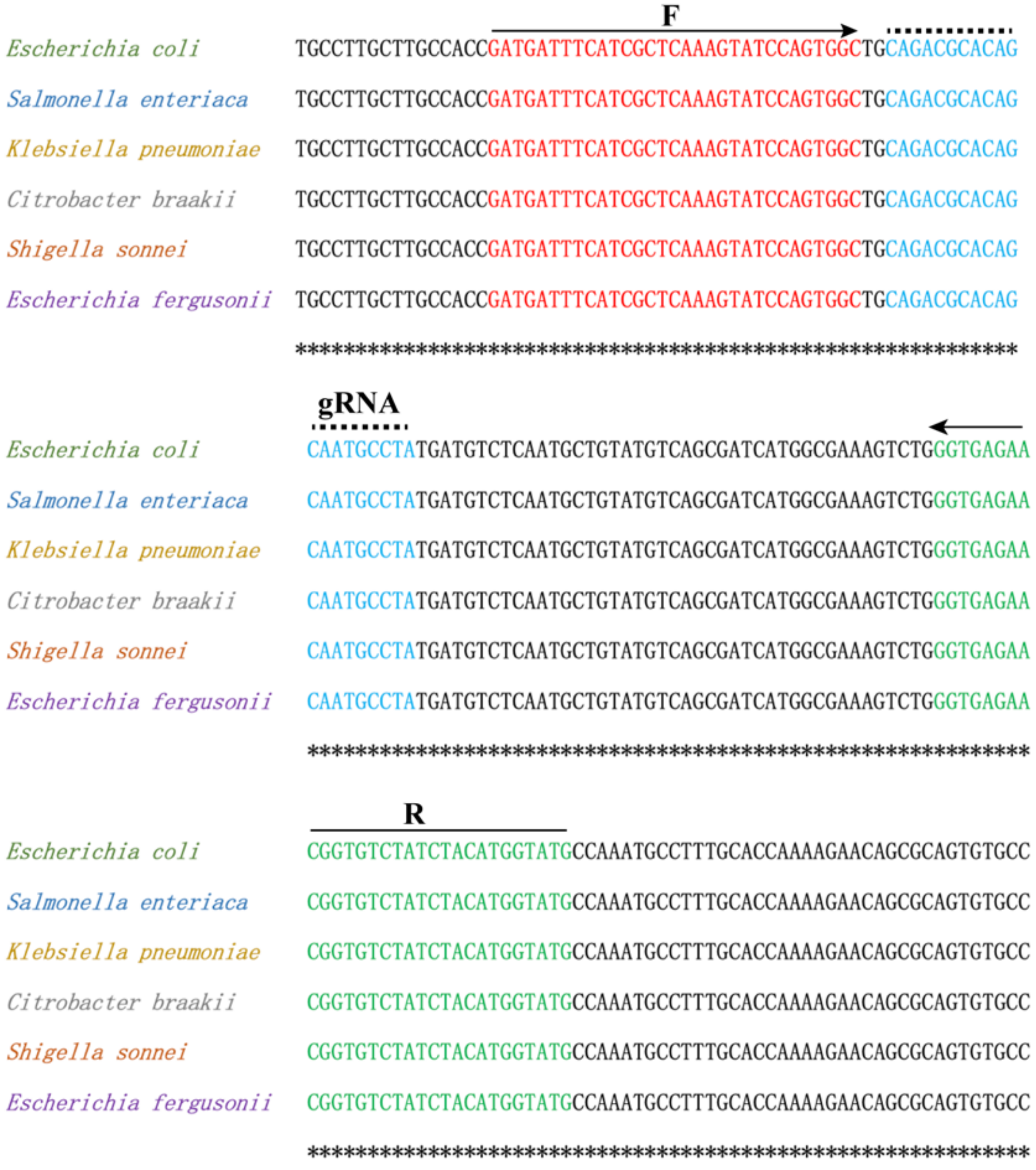

Supplement: Supplemental file 1 — Download spectrum.01884-22-s0001.pdf, PDF file, 0.8 MB [file spectrum.01884-22-s0001.pdf]
